# Supplementary material for: Experimental Characterization of the Pyridine:Acetylene Co-crystal and Implications for Titan’s Surface
Source: ACS Earth Space Chem. 2023 Feb 28;7(3):597–608. doi: 10.1021/acsearthspacechem.2c00377 (PMC10026175; doi:10.1021/acsearthspacechem.2c00377)
Supplement: Supplementary file 1 — sp2c00377_si_001.pdf [file sp2c00377_si_001.pdf]

Experimental Characterization of the Pyridine:Acetylene Co-crystal and Implications for Titan's Surface

Ellen C. Czaplinski, Tuan H. Vu, Morgan L. Cable, Mathieu Choukroun, Michael J. Malaska, Robert Hodyss*

NASA Jet Propulsion Laboratory, California Institute of Technology, 4800 Oak Grove Dr, Pasadena, California 91109, United States

*Corresponding author. Email address: ellen.c.czaplinski@jpl.nasa.gov (Ellen C. Czaplinski)

KEYWORDS co-crystalline, hydrocarbon, Raman spectroscopy, powder X-ray diffraction, molecular mineral

Supporting Information

6 pages, 4 figures, 1 table,

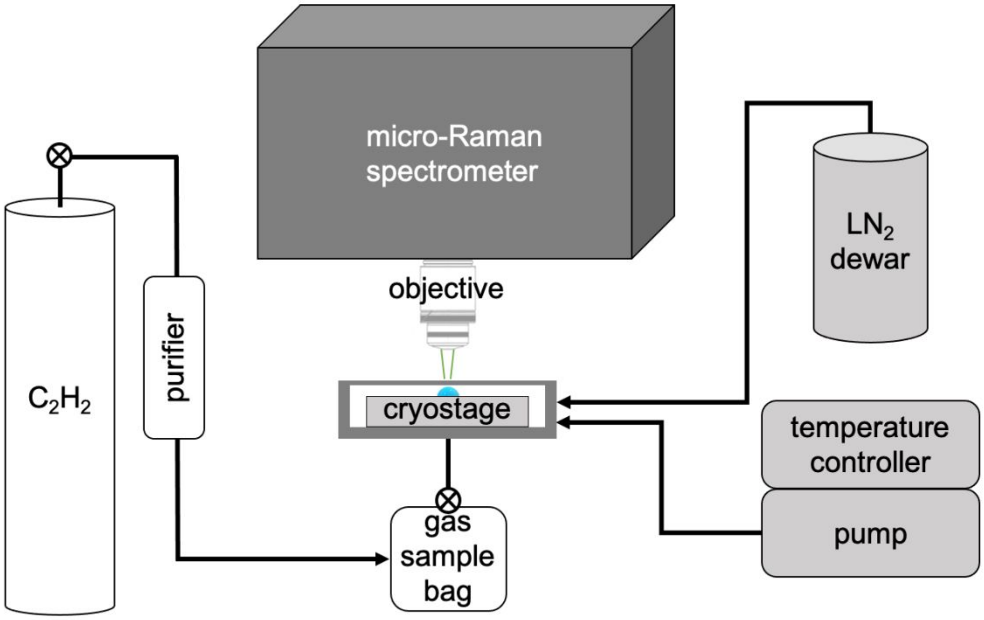

Figure S1. A schematic diagram of the micro-Raman experimental setup. Note the aliquot of liquid pyridine (denoted by blue half-sphere within the cryostage) that is deposited directly onto the slide within the cryostage.

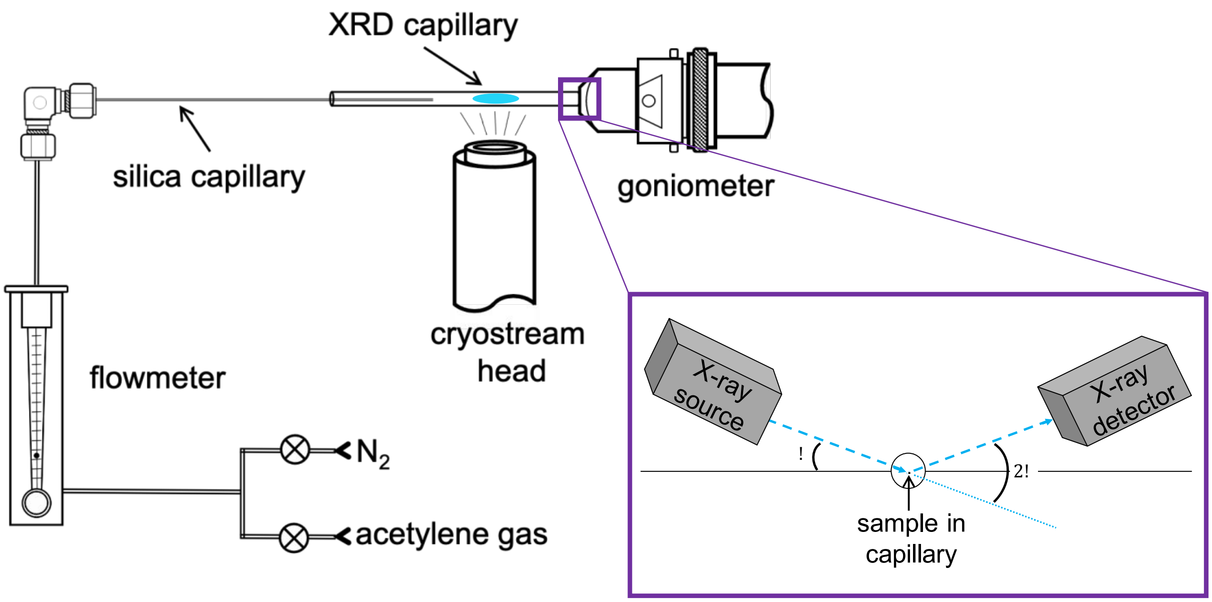

Figure S2. A schematic diagram of the X-ray diffractometer (XRD) setup depicting the custom-built gas introduction system. Note the co-crystal sample (denoted by blue oval within the XRD capillary). Inset: a front-facing view of the XRD showing the relationship between the X-ray source and the X-ray detector with regard to the sample. This figure was modified from Hodyss et al. (2021) with permission of the International Union of Crystallography, according to the terms and conditions of use of material published by the International Union of Crystallography.

Table S1. Experimental Raman Shifts of the Lattice Vibrational Modes after Co-crystal formation (90 K), Compared to Reported Raman Band Centers for Pure Acetylene, Pure Pyridine, and the Acetylene Clathrate Lattice Modes (90 K).

Raman Shift (cm^{-1})					
Reported	Pure Acetylene	Pure Pyridine	Co-Crystal	Acetylene Clathrate	$\Delta\nu$ between pure and co-crystal
	81.9				
83[44]		87.1	91.4	91.3	
		101.8	**98.7**		**-3.1**
			**115.4**		
			**121.7**		
		128.9	**135.7**		**6.8**
174.5[42]	163.2			228.2	
			**199.4**		
			**410.9**		

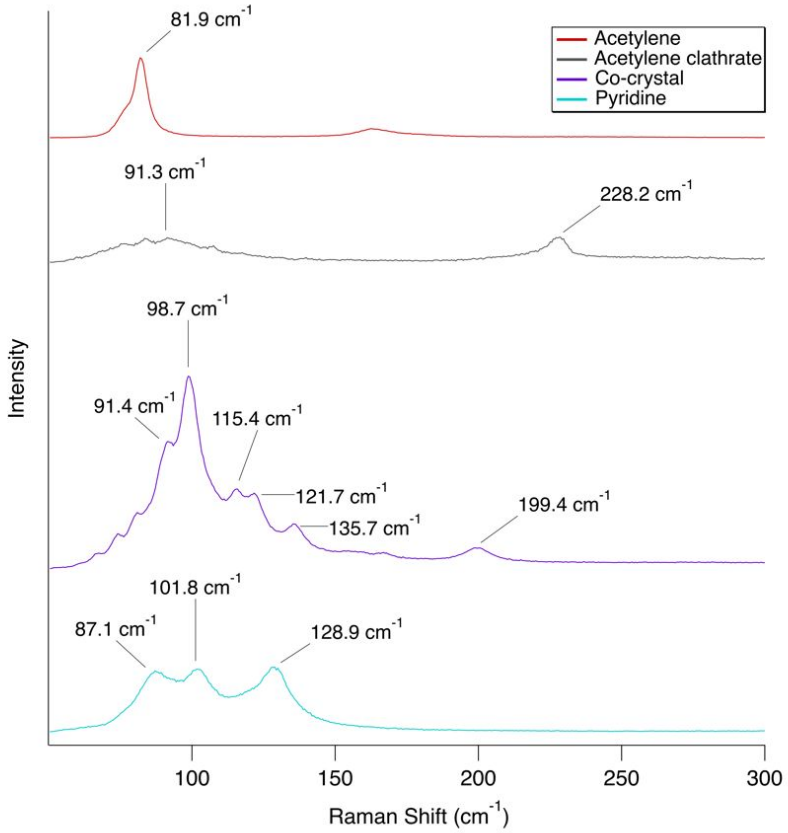

Figure S3. Inset of spectra from Figure 1 showing the lattice vibrational modes of solid acetylene (red, 2× scale), the acetylene clathrate (gray, 8× scale), the pyridine-acetylene co-crystal (purple, 1.5× scale), and solid pyridine (blue, 4x scale).

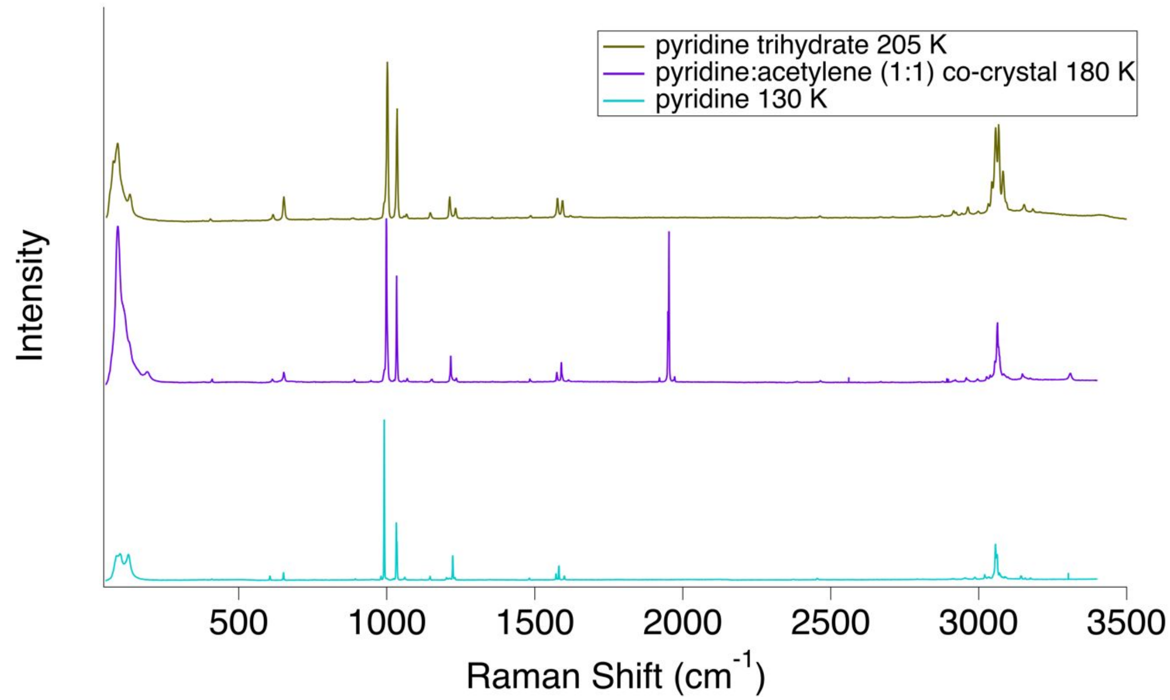

Figure S4. High-resolution Raman spectra of the pyridine trihydrate (gold color), the pyridine:acetylene (1:1) co-crystal (purple), and pure pyridine (cyan; 1.5× scale). Notice the downward slope of the pyridine trihydrate spectrum from ~3200 to 3500 cm^{-1}, which indicates water ice bounded in the trihydrate. The pyridine trihydrate spectrum shows notable differences in spectral shape and blue-shifting in the lattice mode region, the C-C ring stretch/ring bending modes, and the C-H stretching region.
